# Evaluation of Ionic Liquids and Ionic Liquids Active Pharmaceutical Ingredients Inhibition in Elastase Enzyme Activity

**DOI:** 10.3390/molecules26010200

**Published:** 2021-01-02

**Authors:** Fátima A. R. Mota, Sarah A. P. Pereira, André R. T. S. Araujo, M. Lúcia M. F. S. Saraiva

**Affiliations:** 1 LAQV, REQUIMTE, Laboratory of Applied Chemistry, Department of Chemical Sciences, Faculty of Pharmacy, Porto University, Rua de Jorge Viterbo Ferreira, 228, 4050-313 Porto, Portugal; fatimaamota@gmail.com (F.A.R.M.); sarah.pp@hotmail.com (S.A.P.P.); andrearaujo@ipg.pt (A.R.T.S.A.); 2 Unidade de Investigação para o Desenvolvimento do Interior, Instituto Politécnico da Guarda, Av. Dr. Francisco de Sá Carneiro, No. 50, 6300-559 Guarda, Portugal

**Keywords:** ionic liquids, ionic liquids active pharmaceutical ingredients, enzyme activity, elastase, inhibition

## Abstract

Human neutrophil elastase (HNE) is used as diagnostic biomarker for inflammation/infection. In this work, 10 ionic liquids (ILs) and 11 ionic liquids active pharmaceutical ingredients (ILs-APIs) were tested to evaluate the inhibition effect on the activity of porcine pancreatic elastase enzyme, frequently employed as a model for HNE. The insertion of ionic liquids in some drugs is useful, as the insertion of ILs with inhibitory capacity will also slow down all processes in which this enzyme is involved. Therefore, a spectrophotometric method was performed to the determination of EC_50_ values of the compounds tested. EC_50_ values of 124 ± 4 mM to 289 ± 11 mM were obtained, with the most toxic IL for elastase being tetrabutylammonium acetate and the least toxic 1-butyl-3-methylimidazolium acetate. Moreover, sodium salicylate (raw material) presented the lower and benzethonium bistriflimide the higher EC_50_ when compared with all the IL-APIs tested. This work provides significant information about the effect of the studied IL and IL-APIs in elastase enzyme activity.

## 1. Introduction

Human neutrophil elastase enzyme (HNE) is one of the most abundant neutral proteinases and is produced by neutrophilic granulocytes [1]. The neutrophilic granulocyte is the first nucleated cell to infiltrate the wound bed after the integrity of the skin has been disrupted.

In inflammatory sites, there are massive and constant infiltrations of polymorphonuclear neutrophils (PMNs) and the enzyme HNE is abundantly released into the intracellular space, activating pro inflammatory mediators and recruiting more neutrophils [2,3,4]. HNE is able to degrade a variety of structural and functional proteins deposited in wounds such as collagen and fibronectin, as well as key growth factors such as tumor necrosis factor a (TNF-a) [1,5]. However, the excessive production of HNE and other proteolytic enzymes by PMN granulocytes leads to extensive pathological tissue destruction in a number of disorders, including delayed and chronic wound healing [1]. 

Wound infection is a severe complication during wound healing causing diagnostic and therapeutic problems [1,6]. The infection is characterized by excessive neutrophil stimulation, resulting in the release of proteolytic enzymes such as HNE in plasma [7,8]. HNE has a high activity in wound healing only for a few days. Thus, in non-healing wounds, the activity of this enzyme remains high for very long time, leading to the degradation of the reconstituted tissue and often results in wound infection. This enzyme is found since the infection begins, so its activity can be used as a diagnostic marker for the detection of wound inflammation and infection [1,9].

Since elevated HNE levels are described at the very beginning of infection, allows chronic wounds to be treated early before obvious clinical signs of infection are present. Measurement of serum, plasma and urinary elastase is often used as an indicator of the severity of inflammatory disease. However, the presence of neutrophilic enzymes can also be measured in wound fluid, providing early warning of wound infection [1].

As this is a human enzyme present since the onset of infections and inflammation, it is favorable to develop methods to inhibit it preventing the progression to more severe states of infection or inflammation.

Ionic liquids (ILs) are defined as organic salts formed by cations and anions with a melting point below 100 °C and they present interestingly physical properties such as extremely low vapor pressure, wide liquid temperature range, no inflammability, and high thermal and chemical stability [10].

There are several factors inherent in IL that can lead to enzymatic inhibition such as polarity, viscosity, nucleophilicity, hydrophobicity, enzyme dissolution, surfactant effect, anion, cation and alkylated side chain [11]. From the pharmaceutical point of the view, an IL approach in the design of new APIs appears to be appropriate as it enables the chemical manipulation of the compounds with specific objectives related to the manufacturing process, the stability of the formulations, bioavailability and eventual adverse effects [12,13]. An IL approach in the design of new APIs seem to be suitable as it allows a huge number of viable cation-anion combinations while providing singular properties inaccessible in solid salts, namely improved solubility and absence of polymorphic forms [14,15]. The research in this field has been focused mostly on the synthesis of new IL-APIs, their physico-chemical characterization and in vitro assessment of the expected pharmacological activity [16,17].

In this work, different ILs and ionic liquids active pharmaceutical ingredients (ILs-APIs) were tested to the ability to inhibit porcine elastase enzyme activity. Porcine elastase and HNE belongs to the same protease family and they present the same catalytic center, being used several times as a model protein for HNE [18].

The negative effect that ILs can have on enzyme reactions is advantageous when it is intended to inhibit enzyme activity, stability or structure. The association of ionic liquids with some drugs (ILs-APIs) or even its use as a drug is advantageous in this regard as it can have a possible healing effect by impairing elastase activity. On the other hand, ILs can be good alternatives as drug solvents as their toxicity in the human organism is usually lower than that which organic solvents can develop [11].

In order to evaluate the effect of these compounds (ILs and IL-APIs), an enzymatic reaction based on the conversion of the substrate N-succinyl-Ala-Ala-Ala-p-nitroaniline to succinyl-Ala-Ala-Ala and p-nitroaniline by the enzyme elastase was performed. The enzyme activity is determined by the formation of p-nitroaniline, the chromogenic compound resulted from the reaction. Substrate degradation was continuously monitored in the spectrophotometer by measuring increasing absorbance at 405 nm.

## 2. Results and Discussion

The methodology followed to evaluate elastase enzyme activity and its subsequent inhibition by ILs and IL-APIs was optimized regarding the temperature, reaction time, substrate concentration, and enzyme volume and concentration, as described in the Materials and Methods.

### 2.1. Evaluation of Ionic Liquids Inhibition in Elastase Enzyme Activity

After establishing the conditions of the enzyme inhibition assay, the effect of ten commercially available ILs on Elastase activity was evaluated. The tested compounds showed variable chemical structures and incorporated different structural elements, allowing the identification of possible fractions that could act as toxophores (Table 1).

The enzyme activity was studied in aqueous medium and ILs were prepared daily in aqueous solutions with increasing concentrations.

The EC_50_ values for each IL were calculated as the inhibitory concentration causing a 50% decrease in Elastase activity [19]. The EC_50_ values were automatically calculated, using GraphPad prism8 software.

**Figure 1:**
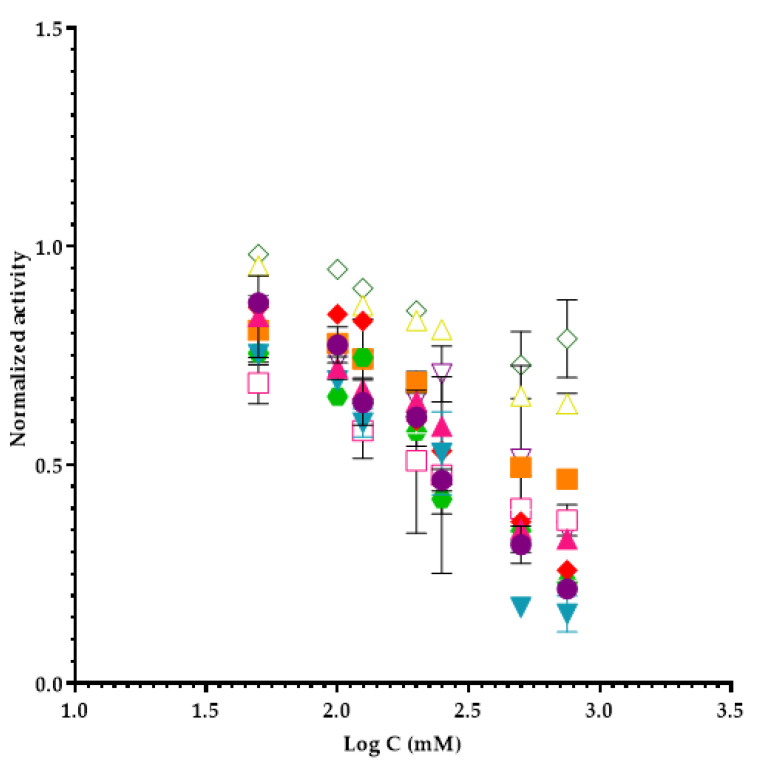
Inhibition profiles of ILs in elastase assays. Subtitles: ● 1-butyl-3-methylimidazolium tetrafluoroborate; **▲** 1-ethyl-3-methylimidazolium tetrafluoroborate; **▼** 1-butyl-3-methylimidazolium chloride; **♦** 1-ethyl-3-methylinidazolium trifluoromethanesulfonate; **⬣** 1-butyl-4-methylpyridinium tetrafluoroborate; **■** tetrabutylammonium chloride; **☐** tetrabutylammonium acetate; **△** tetrabutylphosphonium methanesulfonate; **▽** 1-butyl-3-methylinidazolium acetate; **◇** 1-butyl-3-methylpyrrolidinium-chloride.

The studied ILs, and the respective calculated EC_50_ values are shown in Table 1, and the inhibition profile of each one is represented graphically in Figure 1.

Looking to the results, we can conclude that the most toxic IL for elastase is tetrabutylammonium acetate and the least toxic is 1-butyl-3-methylimidazolium acetate.

ILs offer several ranges of physical and chemical properties, depending on cations and anions. These physico-chemical properties (such as polarity, hydrophobicity, viscosity, etc.) of ILs play an important role in enzyme activity and stability. For example, considering lactic dehydrogenase (LDH) enzyme, the anions influence its activity in the following order TfMs > BF4 > Br > Cl [20]. According to the results, more hydrophobic ILs have more affinity to the active loop of LDH enzyme, which is the main driving force for the ILs interaction with LDH and for instance also with lipase [21].

The toxicity decrease of the ILs may be explained by a decrease in the value of the octanol–water partition coefficient (K_ow_) by the introduction of an oxygenated group, which results in lower lipophilicity and consequently lower toxicity. Moreover, the position of the oxygen atom in the side chain also influences the toxicity of the compound, as it causes a change in the charge distribution thorough the chain and so alterations in the hydrophobicity of the molecule and consequently its toxicity [22].

Furthermore, there are several factors that can interfere with the toxicity of an IL, such as the length of the side chain of alkyl cations, the presence of a functionalized side chain in the cation, the anionic portion and the cationic portion of the IL.

The anion of IL impacts the enzyme stability and activity through its ability to form hydrogen bonds and nucleophilicity properties, as demonstrated by several groups. What is generally observed is that an anion with a high hydrogen bonding capacity interacts strongly with the enzyme, causing a conformational change in the structure and affecting its activity [23].

Thus, it was important to evaluate the influence of different anions on the activity of the enzyme elastase, using tetrafluoroborate (BF_4_), chloride (Cl), trifluoromethanesulfonate (Tfms) and acetate (Ac) nuclei. So, we can compare ILs with different anions and cations to understand which are the most toxic for this enzyme and how it is influenced by them.

Comparing the ILs [bmim] BF4 (EC_50_ = 208 ± 8 mM), [bmim] Cl (EC_50_ = 248 ± 49 mM), and [bmim] Ac (EC_50_ = 289 ± 22 mM), we can conclude that the BF_4_ anion is the most toxic for the elastase enzyme since it has a lower EC_50_ value. Lower EC_50_ values were observed for ILs with the BF4 anion such as ([bmim] BF_4_ (EC_50_ = 208 ± 8 mM) and [emim] BF_4_ (EC_50_ = 201 ± 51 mM) compared to other anions. This anion has previously shown considerable negative effects on other enzymes such as acylase [24] and cytochrome C oxidase [25] and even microorganisms [23]. The nucleophilic nature alters the conformation of the enzymes by interacting with the positively charged sites in their structures [26]. The same happens when we compare the ionic liquids [emim] BF_4_ (EC_50_ = 201 ± 51 mM) and [emim] Tfms (EC_50_ = 239 ± 5 mM), where the BF_4_ anion is also more toxic. One possible explanation may be that molecules that contain fluorine on their composition can be hydrolsed and produce toxic hydrofluoric acid [24].

The ILs composed with the anions [emim] Tfms (EC_50_ = 239 ± 5 mM) and [Tbph] Ms (EC_50_ = 229.9 ± 0.6 mM) and [bmim] Ac (EC_50_ = 289 ± 22 mM) revealed an insignificant effect on the enzymatic behaviour and, therefore, we conclude that the ihibition produced by these ILs is more related to the cationic group [25]. The low inhibition effect of these anions has also been verified for other enzymes and organisms: cytochrome C oxidase, carboxylesterase, catalase and V. fischeri [25]. On the other hand, the Ac anion demonstrated in another case a high inhibition, when combined with the ammonium cation [tba] Ac (EC_50_ = 176 ± 6 mM). This could possibly be explained by the combination of this anion with the cation that causes high inhibition.

The studied ILs can be divided into two groups: one comprises non-aromatic ILs, based on piperidinium and pyrrolidinium, while the other comprises aromatic ILs based on imidazole and pyridinium [27].

Regarding the cationic head nucleus, it has been described by several authors as one of the main structural elements that contribute to the toxicity of ILs.

Therefore, the influence of different cations on elastase activity was assessed using the nuclei: imidazolium (im), pyridinium (pyr), pyrrolidinium (pyrr) and phosphonium (ph) [28]. The strongest inhibition was obtained with ILs containing positively charged nitrogen pyrrolidinium (bmpyrr) Cl (EC_50_ = 194 ± 1 mM), imidazolium [emim] BF_4_ (EC_50_ = 201 ± 51 mM) and [bmim] BF_4_ (EC_50_ = 208 ± 8 mM)] compared to other cations as [bmpy] BF_4_ (EC_50_ = 239 ± 3 mM) and [tba] Cl (EC_50_ = 233 ± 24 mM). Except [bmim] Ac (EC_50_ = 289 ± 22 mM) which is a cation imidazole with the influence of an Ac anion, decreases the toxicity of the imidazolium group. This only happens for elastase, since these groups have, in general, high toxicity.

The IL with pyrrolidinium (bmpyrr) Cl (EC_50_ = 194 ± 1 mM) as a cationic nucleus structure, slightly inhibited the enzyme comparing with the imidazolium analog (bmim) Cl (EC_50_ = 248 ± 49 mM) [29]. The pyrrolidinium groups (bmpyrr) Cl (EC_50_ = 194 ± 1 mM) showed lower EC_50_ values than any IL based on aromatic imidazolium, so ILs containing pyrrolidinium groups are more toxic for elastase enzyme than the imidazolium core [25].

Among the cations that incorporate an aromatic ring, the group of the imidazolium core (bmim) BF_4_ (EC_50_ = 208 ± 8 mM) exhibited a greater toxic effect than the pyridinium (bmpy) BF_4_ (EC_50_ = 239± 3 mM). Although there is no consensus on the correlation between these two groups in the literature, a similar trend has been described for the enzyme acetylcholinesterase [25].

The effect of the length of the alkylated side chain has been discussed by several authors as responsible for an increase in toxicity [30]. This effect was named “side chain effect”, that is, the elongation of the alkylated side chain of IL increases its inhibition [19,31]. Moreover, the lipophilicity impact in the inhibition of ILs was claimed by some authors, demonstrating that the length of the alkyl side chain as the main determinant in its modulation [28,32]. So, the intrinsic inhibition of the side chain was evaluated by increasing its size (2 and 4 carbons) in the imidazolium based-ILs [25]. This is not verified in the results obtained, since if we observe the ILs (emim) BF_4_ (EC_50_ = 201 ± 51 mM) and (bmim) BF_4_ (EC_50_ = 208 ± 8 mM), we have 2 and 4 carbons respectively, and we have a more toxic effect in the IL with the lower alkylated side chain. The insertion of two more carbons in the alkyl side chain to get bmim (BF4) was not enough to influence the toxicity of the IL. In this case, probably the lipophilicity of this two ILs is relatively low and there are other factors leading the potential inhibition of these compounds as a better accessibility to the active center of the enzyme.

Despite the differences between the EC_50_ being slight, this can be justified, considering that it has already been described for other enzymes, such as acylase, that the harmful effect of the alkylated side chain was not as noticeable as expected [19,24]. The increase in the inhibition of an IL with the increase of the alkylated side chain can only be observed sharply from the 4 carbons. This may explain the results obtained [33]. Since the difference between the EC_50_ is very low, we can conclude that the length of the side chain of alkyl cations is significantly variable.

The influence of the cation and the anion on the inhibition of ILs seems to be independent, since the inhibition is the result of the sum of both impacts of cations and anions, not forgetting the external factors.

### 2.2. Evaluation of Ionic Liquids Active Pharmaceutical Ingredients (IL-APIs) Inhibition in Elastase Enzyme Activity

In the same conditions, we also tested several IL-APIs compounds. As it was explained before, ILs used as pharmaceuticals present new and unique properties compared to solid dosage forms.

**Figure 2:**
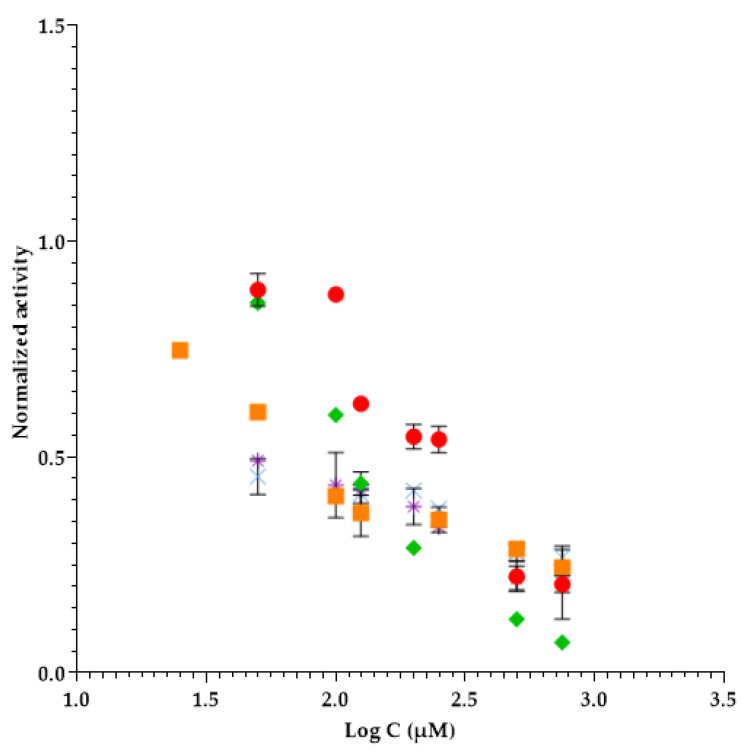
Inhibition profiles of IL-APIs in elastase assays. Subtitles: ● Lithium bistriflimide; ✴ Benzalkonium salicylate; ■ Sodium salicylate; × 1-ethyl-3-methylimidazolium salicylate; ♦ Benzethonium chloride.

So, in Table 2, we present the IL-APIs tested that present an inhibitory effect to elastase enzyme and the inhibition profiles of each one is represented graphically in Figure 2.

Considering Table 2 and comparing with ILs results, we can see that IL-APIs EC_50_ are lower than ILs. So, it is necessary a lower concentration to obtain an inhibitory elastase enzyme effect using these IL-APIs.

The IL-API with lower EC_50_ in elastase activity was sodium salicylate (124 ± 4 µM) and the IL-API that presents higher EC_50_ value was benzethonium bistriflimide (>1000 µM).

**Figure 3:**
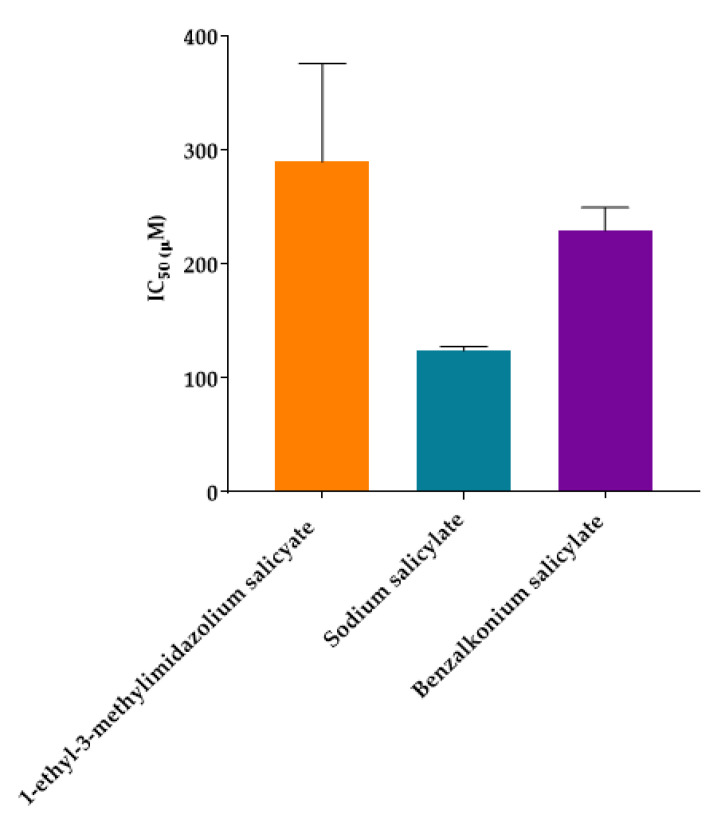
ANOVA statistic test for salicylate anion IL-APIs.

To better understand the results obtained, an ANOVA statistic test was performed for all the IL-APIs that present salicylate anion in their chemical constitution (Figure 3).

According to Figure 3, it is possible to verify that sodium salicylate (raw material) is more toxic than the IL-APIs with the same anion. According to the EC_50_ values (sodium salicylate EC_50_ = 124 µM, 1-ethyl-3-methylimidazolium salicylate EC_50_ = 290 µM and benzalkonium salicylate EC_50_ = 230 µM), 1-ethyl-3-methylimidazolium salicylate and benzalkonium salicylate are 2.3 and 1.8 times less toxic than sodium salicylate, respectively. As stated by ANOVA statistic test, even with these differences in EC_50_ values of these three compounds, there are no significant differences between them (*p* > 0.05).

The bistriflimide anion reduces the inhibition of the IL-APIs when combined with benzethonium and phosphonium cations when compared with the corresponding starting materials incorporating the chloride anion. These results are supported by the previous report, where it is suggested that the cation type combined with bistriflimide anion can circumvent its negative effect and in some cases the IL inhibition can be reduced [34].

In a pharmaceutical perspective, salicylate IL-APIs with distinct cations present antiseptic and antibacterial activity, namely benzalkonium (only the 1-ethyl-3-methylimidazolium cation is neutral), combined with the salicylate anion, with well recognized analgesic and antipyretic activity [35]. So, as sodium salicylate presents a lower EC_50_, it could present a higher inhibition for elastase enzyme and at the same time an analgesic effect.

To better understand the action of IL-APIs in elastase, we compare the EC_50_ values obtained for carboxylesterase (other enzyme) and *V. fischeri* (a microorganism) [36].

Comparing the values obtained, we can see that 1-ethyl-3-methylimidazolium salicylate is the only IL-API that the EC_50_ is lower for elastase than for carboxylesterase and *V. fischeri* is. So, this IL-API presents a higher toxicity for elastase than in carboxylesterase and *V. fischeri*.

When we compare the EC_50_ values of sodium salicylate and benzethonium chloride for elastase (124 ± 4; 166 ± 4) and carboxylesterase (116.99 ± 0.02; 123.700 ± 0.009) enzymes, we can conclude that the inhibition effect is similar for them, because the EC_50_ values are also resemble.

On the other hand, the other IL-APIs that were tested in elastase, carboxylesterase and *V. fischeri* activity (benzalkonium salicylate, trihexyltetradecylphosphonium docusate, benzethonium salicylate, benzethonium docusate, benzethonium bistriflimide, and trihexyltetradecylphosphonium chloride) the EC_50_ values obtained for elastase are higher than for carboxylesterase and *V. fischeri* [36].

As in ILs, the K_ow_ is an important parameter to evaluate the inhibition of ILs-APIs. In literature, there are already some K_ow_ defined for some ILs-APIs. Pinto et al. determined the partitioning properties of some salicylate IL-APIs [Cetylpyridinium salicylate (CetPySal), benzethonium salicylate (BeSal) and 1-ethyl-3-methylimidazolium salicylate (emimSal)] by the calculation of partition coefficients (K_p_), by derivative spectroscopy, using the K_p_ calculator. All the tested IL-APIs present higher partition between the aqueous and lipid phases. This is confirmed by the significant increase of the K_p_ values compared with the corresponding starting materials (sodium salicylate (NaSal), cetylpyridinium chloride (CetPyCl), benzethonium chloride (BeCl), 1-ethyl-3-methylimidazolium chloride (emin Cl)) that incorporate either the anion or the cation of each IL-APIs. The obtained K_p_ values were up to about 6 times higher than those of the corresponding anions or cations [35].

As a positive control, it was tested a potent HNE inhibitor named MeOSuc-Ala-Ala-Pro-Val-chloromethylketone. The EC_50_ value obtained was 0.47 mM ± 0.05. Comparing this compound with the others tested, we can conclude that the HNE inhibitor presents a lower EC_50_ for elastase than all ILs and higher than IL-APIs.

## 3. Materials and Methods

### 3.1. Reagents

All solutions were prepared using ultrapure water from a Milli-Q water plus system with specific conductivity of less than 0.1 µS cm^−1^ and the chemicals of analytical grade.

Elastase from porcine pancreas, N-Succinyl-Ala-Ala-Ala-p-nitroanilide, HEPES sodium salt, sodium acetate, sodium hydroxide and acetic acid were purchase to Sigma-Aldrich^®^ (St. Louis, Missouri, United States). Sodium chloride and dimethyl sulfoxide (DMSO) were purchase to Merck^®^ (Darmstadt, Germany) and Uvasol^®^ (Darmstadt, Germany), respectively.

A HEPES buffer solution 0.1 M with sodium chloride (NaCl) 0.5 M at pH 7.5 was used to dissolve the reagents. The buffer solution was prepared weighting 14.6 g of sodium chloride and 12.4g of HEPES and dissolving in about 150 mL of water. The pH 7.5 was adjusted with a 1 M NaOH solution, and final volume was adjusted to 500 mL. A sodium acetate buffer (pH 5.5) solution was used to dissolve the enzyme. The buffer solution was prepared weighting 1.64 g of sodium acetate and dissolving in about 50 mL of water. The pH 5.5 was adjusted with a 17.4 M acetic acid solution, and final volume was adjusted to 100 mL.

All the enzyme elastase porcine was reconstituted by stock solution of 1 U mL^−1^ using 2.5 mg of powder enzyme in 20 mL of sodium acetate buffer (pH 5.5). The stock solution was divided into 14 eppendorfs with a total volume of 1.43 mL per eppendorf. The eppendorfs were frozen.

A solution of *N*-Succinyl-Ala-Ala-Ala-p-nitroanilide was prepared daily dissolving 11.25 mg of substrate in 10 mL of DMSO.

For enzyme inhibition assays the aqueous solutions of: 1-Butyl-3-methylimidazolium tetrafluoroborate (bmim [BF_4_]), 1-Butyl-3-methylimidazolium chloride (bmim [Cl]), 1-Ethyl-3-methylimidazolium tetrafluoroborate (emim [BF_4_]), 1-Ethyl-3-methylimidazolium trifluoromethanesulfonate (emim [TfMs]), 1-Butyl-1-methylpyrrolidinium chloride (bmpyr [Cl]), 1-Butyl-4-methylpyridinium tetrafluoroborate (bmpy (BF_4_)), Tetrabutylammonium chloride (tba (Cl)), Tetrabutylammonium acetate (tba (Ac)), Tetrabutylphosphonium methanesulfonate (tbph (ms)), 1-Butyl-3-methylimidazoliumacetate (bmim (Ac)), were prepared daily. ILs were purchased from Sigma-Aldrich^®^ and kept in anhydrous environment.

The ionic liquids active pharmaceutical ingredients (ILs-APIs) were synthesized according to [35,36] and the aqueous solutions of lithium bistriflimide, 1-ethyl-3-methylimidazolium salicylate, benzethonium chloride, sodium salicylate, benzalkonium salicylate, trihexyltetradecylphosphonium docusate, benzethonium salicylate, benzethonium docusate, benzethonium bistriflimide, tributylmethylphosphonium salicylate, and trihexyltetradecylphosphonium chloride.

The chemical structures of all ILs and IL-APIs are presented in Supplementary Materials.

The HNE inhibitor named MeOSuc-Ala-Ala-Pro-Val-chloromethylketone was purchased from BioVision^®^ (San Francisco Bay).

### 3.2. Apparatus

Absorbance measurements were performed on a Jasco^®^ V-660 dual-beam spectrophotometer (Hachioji-shi, Tokyo) between 220 and 500 nm using 1 cm optical path quartz cells.

### 3.3. Optimization of Elastase Porcine Reaction

There is often a variety of different assays methods available for an enzyme. Taking this into account, there are some assays methods for elastase. So, the methodology developed in this work was based on the protocols described by Schulz-Fincke et al. and Ferreira et al. [9,37].

However, some optimization was done using the univariant method, which consists of changing, within a certain interval, the parameter to be optimized keeping the remaining parameters fixed. First, some physical and chemical factors that affect the reaction development were evaluated.

The pH of the reaction medium is an extremely important factor in the case of enzymatic reactions, directly influencing the sensitivity of the reaction. Also, changes in pH change the shape of the enzyme active site. Each enzyme has an optimal acting pH, where its activity is maximum. So, HEPES buffer at pH 7.5 was used to ensure that the pH was controlled during the reaction.

For the optimization it was keep a substrate volume (10 μL) fixed in all assays, changing only its concentration. Regarding the enzyme, once it was reconstituted, changing the volume we put in each eppendorf also changed its concentration, with 1 U (units) of enzyme (concentration) corresponding to 1.43 mL of the volume of the enzyme.

The optimized parameters comprised the temperature, reaction time, substrate concentration and enzyme concentration and volume (Table 3).

The temperature is an important parameter to consider during an enzymatic reaction, as each enzyme has its optimal temperature which when exceeded can cause it to be inactive or changes in its active center. Moreover, the temperature effect on enzyme activity has been defined by two well-proved thermal parameters: the Arrhenius activity energy, which consists in the temperature effect on the catalytic rate constant (ƙ_cat_); and thermal stability, which defines the temperature effect on the thermal inactivation rate constant (k_inact_) [38]. For elastase, it was fixed at 37 °C where higher absorbance signal was obtained.

Regarding the reaction time, 10, 15, and 20 min were evaluated. For 20 min, when we increased the enzyme concentration, the absorbance remains the same. This means that the substrate has already been completely converted into the product after 20 min, regardless the enzyme concentration used. On the other hand, for 10 and 15 min, we can saw that the absorbances are similar. So, as a compromise between sensitivity and the sampling rate, the reaction time selected was 10 min.

Regarding substrate concentration, it was tested three different concentrations: 50 µM, 75 µM and 100 µM. When the substrate concentration has been increased from 50 µM to 100 µM, the absorbance has also doubled (0.2 UA to 0.5 UA). So, the substrate concentration selected was 100 µM, since it is favourable to have high absorbance values in order to calculate the percentage of inhibition in presence of ILs and ILs-APIs.

Regarding the enzyme concentration/volume, there is an increase in absorbance up to 30 μL of enzyme, from then on, between 30 μL and 50 μL the absorbance remained similar. Thus, considering the sensitivity–consumption ratio of reagents, it was decided to select as enzyme volume 30 μL of enzyme (corresponding to 0.0209 U), since it is still in a linear zone and with a high absorbance.

After all the parameters optimized, the calibration curve obtained was: A = (0.006 ± 0.117) C (µM) + (-0.090 ± 0.001); R^2^ = 0.992 where A is the absorbance obtained in the assay and C is the concentration of substrate in µM, respectively, with 95% confidence limits for the intercept and slope. The detection limit was calculated using the absorbance corresponding to the ordinate value at the origin with the addition of three times the Sy/x, interpolating this value on the calibration curve. The detection limit obtained was 7.06 µM. The quantification limit was calculated through the absorbance corresponding to the ordinate value at the origin plus ten times the Sy/x, interpolating this value on the calibration curve. The limit of quantification obtained was 23.54 µM.

### 3.4. Optimized Batch Procedure

After all the optimization, the final batch procedure was defined.

All the reagents (HEPES buffer, *N*-succinyl-Ala-Ala-Ala p-nitroanilide, Elastase and acetate buffer) were added sequentially in eppendorf, being the final volume equal to 250 µL. So, in each eppendorf it was added 137.5 µL of HEPES buffer, 10 µL of *N*-succinyl-Ala-Ala-Ala p-nitroanilide substrate, 30 µL of elastase, 70 µL of acetate buffer, 2.5 µL of DMSO. In inhibitory assays was 87.5 µL of HEPES buffer, 10 µL of *N*-succinyl-Ala-Ala-Ala *p*-nitroanilide substrate, 30 µL of elastase, 70 µL of acetate buffer, 2.5 µL of DMSO, and 50 µL of IL/IL-APIs.

Next, the eppendorf was placed inside the thermostatic bath at 37 °C for 10 min.

After 10 min, the reaction product was measured in spectrophotometer at 405 nm.

### 3.5. Experimental Procedure

All assays were performed in batch mode. Thus, the different reagents were placed manually in an eppendorf at the desired concentrations and volumes. After, they were placed in a thermostatic bath where the reaction temperature was regulated for the desired period. The succinyl-Ala-Ala-Ala and p-nitroaniline result from the cleavage of the substrate *N*-succinyl-Ala-Ala-Ala-p-nitroaniline by porcine elastase enzyme. The enzyme activity was determined by monitoring the chromogenic compound, p-nitroaniline, at 405 nm. All results of ILs and IL-APIs were obtained in duplicate.

## 4. Conclusions

The development of strategies that allow obtaining new effective chemical compounds that bring advantages in their application has been the focus of the pharmaceutical industry. As such, the use of ILs in pharmaceutical applications has been an area of great interest in recent years due to its advantageous characteristics and properties. The selection of organic cations and biocompatible organic/inorganic anions has been referred to as a possibility for producing non-toxic ILs with advantages over other solvents.

Through this experimental work it was possible to evaluate the inhibition of several ILs and IL-APIs in the inhibition of the activity of elastase enzyme.

The results obtained can be a good contribution with regard to the impact of ILs and IL-APIs in humans. Since there are an elastase human enzyme, with high activity in several diseases and found since the beginning of the infection, its inhibition means the reduction of infection/inflammation and/or treatment of diseases. In this way, the objective is to find solvents, in this case ILs that are slightly toxic to the organism and relatively toxic to the enzyme so that they inhibit its activity, being able to act with antimicrobial effect in wounds.

EC_50_ values of 176 ± 4 mM to 289 ± 11 mM were obtained, with the most toxic IL for the enzyme elastase being tetrabutylammonium acetate and the least toxic was 1-butyl-3-methylimidazolium acetate. In general, a preferential enzymatic inhibition by the BF_4_ anion and containing pyrrolidinium and imidazole groups was observed for the ILs studied, with the pyrrolidinium group inhibiting the enzyme with EC_50_ values as low as 193.8 ± 0.6 mM. On the other hand, the methanesulfonate and trifluoromethanesulfonate and phosphonium groups had less negative effect on enzyme activity.

Moreover, the results showed that the structure of the ILs influences their toxicity, that is, the composition of the cation, the alkyl side chains and the anions. These results can be justified by considerations of structure-activity relationship.

With regard to IL-APIs with antimicrobial effect, we can conclude that sodium salicylate present a lower EC_50_ and benzethonium bistriflimide the higher EC_50_, when compared with all the IL-APIs tested in this study.

MeOSuc-Ala-Ala-Pro-Val-chloromethylketone, a positive control of the inhibition of enzyme activity was also tested, and comparing with IL-APIs tested those have even higher potential to inhibit the enzyme and consequently present a possibility of contributing to the decrease of wound infection.

The results obtained in this work provide significant information about the safety of the studied IL-APIs and must be combined in a more extensive analysis when more information about the biological behavior is available. However, these approaches can be considered valuable screening assays, and from this perspective, the results may contribute to the decision of including these compounds in a higher stage of drug development.

## Figures and Tables

**Table 1:** Results of elastase inhibition by ILs expressed as EC_50_.

Ionic Liquid (IL)	IL Abbreviation	Molecular Weight (g mol^−1^)	EC_50_ (mM) ± SD ^(1)^	Confidence Interval
MeOSuc-Ala-Ala-Pro-Val-chloromethylketone	-	503.0	0.47 ± 0.05	±0.47
1-Butyl-3-methylimidazolium tetrafluoroborate	[bmim] BF_4_	226.0	208 ± 8	±70
1-Ethyl-3-methylimidazolium tetrafluoroborate	[emim] BF_4_	197.9	201 ± 51	±457
1-Butyl-3-methylimidazolium chloride	[bmim] Cl	174.7	248 ± 49	±441
1-Ethyl-3-methylimidazolium trifluoromethanesulfonate	[emim] Tfms	260.2	239 ± 5	±46
1-Butyl-1-methylpyrrolidinium chloride	[bmpyrr] Cl	177.7	194 ± 1	±12
1-Butyl-4-methylpyridinium tetrafluoroborate	[bmpy] BF_4_	237.0	239 ± 3	±30
Tetrabutylammonium acetate	[tba] Ac	301.5	176 ± 6	±55
Tetrabutylammonium chloride	[tba] Cl	277.9	233 ± 24	±216
Tetrabutylphosphonium methanesulfonate	[Tbph] Ms	354.5	229.9 ± 0.6	±5
1-Butyl-3-methylimidazolium acetate	[bmim] Ac	198.3	289 ± 22	±200

(1) Standard deviation.

**Table 2:** Results of elastase inhibition by IL-APIs expressed as EC_50_.

Ionic Liquid Active Pharmaceutical Ingredients (IL-APIs)	Molecular Weight (g mol^−1^)	EC_50_ (µM) ± SD ^(1)^	Confidence Intervals
Lithium bistriflimide	287.1	209 ± 12	±110
1-ethyl-3-methylimidazolium salicylate	248.3	290 ± 86	±774
Benzethonium chloride	448.1	166 ± 4	±40
Sodium salicylate *	160.1	124 ± 4	±33
Benzalkonium salicylate	452.8	230 ± 20	±182
Trihexyltetradecylphosphonium docusate	905.4	>700	-
Benzethonium salicylate	549.7	>500	-
Benzethonium docusate	834.2	>200	-
Benzethonium bistriflimide	692.8	>1000	-
Tributylmethylphosphonium salicylate	621.0	>200	-
Trihexyltetradecyphosphonium chloride	519.3	>100	-

(1) Standard deviation; * It is not an IL-API but a salt.

**Table 3:** Tested conditions and respective results of the optimization.

Parameter Tested	Range	Selected Values
Temperature	25 °C and 37 °C	37 °C
Reaction time	10 min, 15 min and 20 min	10 min
Substrate concentration	50 µM, 60 µM, 75 µM, 100 µM and 150 µM	100 µM
Enzyme volume	12.5 µL, 20 µL, 30 µL and 50 µL	30 µL
Enzyme concentration	0.0874 U; 0.0139 U; 0.0209 U and 0.0349 U	0.0209 U

## Data Availability

Data is contained within the article or supplementary material. The data presented in this study are available in this manuscript.

## Supplementary Materials

The following are available online. Figure S1: Chemical structures of the studied ILs. 1: 1-butyl-3-methylimidazolium tetrafluoroborate (bmim [BF4]); 2: 1-ethyl-3-methylimidazolium tetrafluoroborate (emim [BF4]); 3: 1-butyl-3-methylimidazolium acetate (bmim [Ac]); 4: 1-butyl-4-methylpyridinium tetrafluoroborate (bmpy [BF4]); 5: 1-butyl-3-methylimidazolium chloride (bmim [Cl]); 6: 1-ethyl-3-methylimidazolium trifluoromethanesulfonate (emim [TfMs]); 7: 1-butyl-1-methylpyrrolidiniumchloride (bmpyr [Cl]); 8: tetrabutylphosphonium methanesulfonate (tbph [Ms]); 9: tetrabuthylammonium chloride (tba [Cl]); 10: tetrabutylammonium acetate (tba [Ac]); Figure S2: Chemical structures of the studied IL-APIs. 1: Benzethonium docusate; 2: benzethonium salicylate; 3: Lithium bistriflimide; 4: benzethonium bistriflimide; 5: 1-ethyl-3-methylimidazolium salicylate; 6: benzethonium chloride; 7: sodium salicylate; 8: benzalkonium salicylate; 9: trihexyltetradecylphosphonium docusate; 10: trihexyltetradecylphosphonium chloride; 11: tributylmethylphosphonium salicylate. * *n* = 8, 10, 12, 14, 16, 18.

## Sample Availability

All ILs were purchased from Sigma-Aldrich^®^ and IL-APIs compounds were synthesized by Katharina Schröder.
