# Supplementary material for: Evaluation of Ionic Liquids and Ionic Liquids Active Pharmaceutical Ingredients Inhibition in Elastase Enzyme Activity
Source: Molecules. 2021 Jan 2;26(1):200. doi: 10.3390/molecules26010200 (PMC7796259; doi:10.3390/molecules26010200)

Supplementary material

Figure 1: Chemical structures of the studied ILs. 1: 1-butyl-3-methylimidazolium tetrafluoroborate (bmim [BF4]); 2: 1-ethyl-3-methylimidazolium tetrafluoroborate (emim [BF4]); 3: 1-butyl-3-methylimidazolium acetate (bmim [Ac]); 4: 1-butyl-4-methylpyridinium tetrafluoroborate (bmpy [BF4]); 5: 1-butyl-3-methylimidazolium chloride (bmim [Cl]); 6: 1-ethyl-3-methylimidazolium trifluoromethanesulfonate (emim [TfMs]); 7: 1-butyl-1-methylpyrrolidiniumchloride (bmpyr [Cl]); 8: tetrabutylphosphonium methanesulfonate (tbph [Ms]); 9: tetrabuthylammonium chloride (tba [Cl]); 10: tetrabutylammonium acetate (tba [Ac])

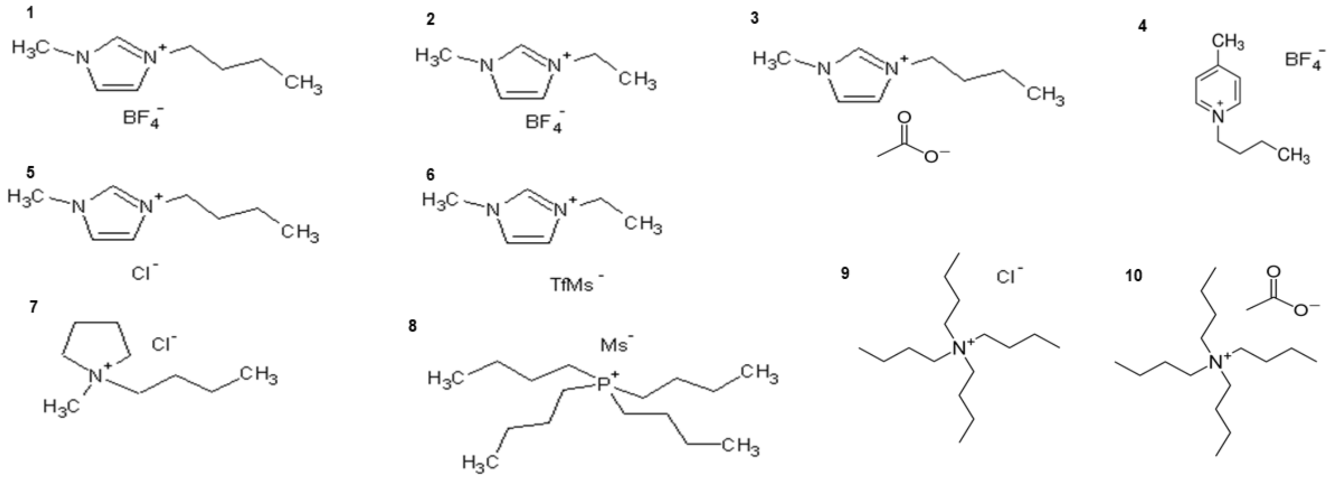

Figure 2: Chemical structures of the studied IL-APIs. 1: Benzethonium docusate; 2: benzethonium salicylate; 3: Lithium bistriflimide; 4: benzethonium bistriflimide; 5: 1-ethyl-3-methylimidazolium salicylate; 6: benzethonium chloride; 7: sodium salicylate; 8: benzalkonium salicylate; 9: trihexyltetradecylphosphonium docusate; 10: trihexyltetradecylphosphonium chloride; 11: tributylmethylphosphonium salicylate.
* n = 8, 10, 12, 14, 16, 18

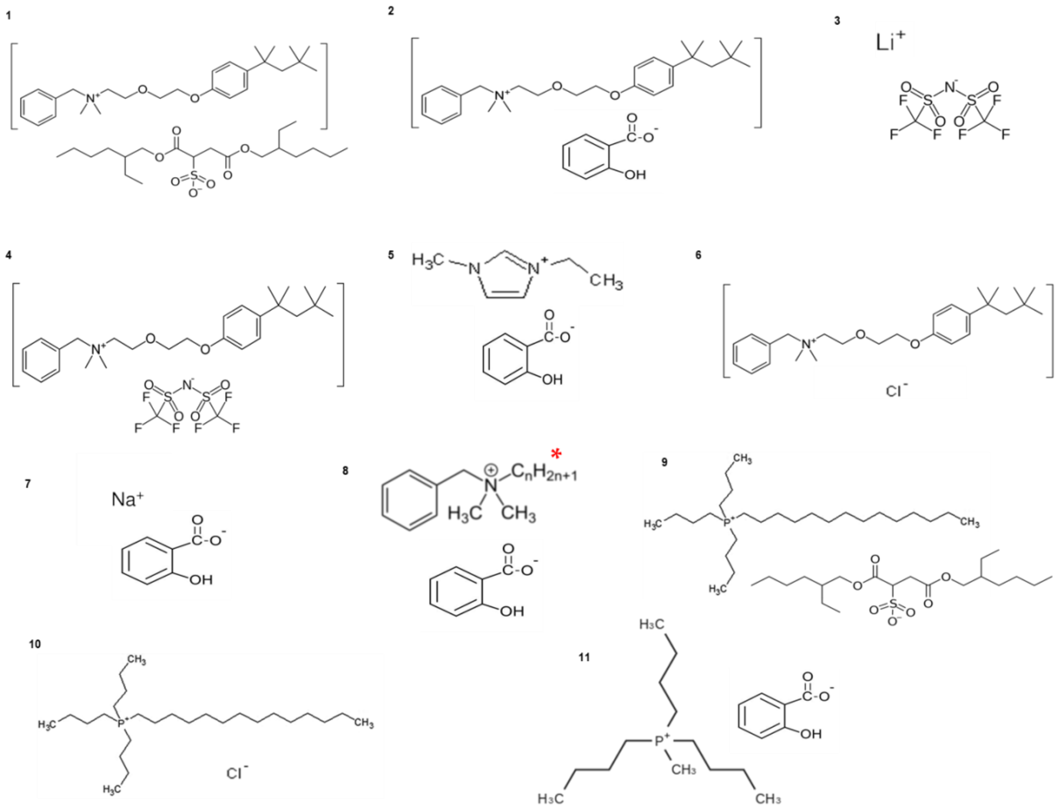

Supplement: Supplementary file 1 [file molecules-26-00200-s001.pdf]
